# A new type of abdominal incision for emergencies in pregnancy: a case report

**DOI:** 10.11604/pamj.2020.37.347.23100

**Published:** 2020-12-15

**Authors:** Hasan Yüksel, Tolga Atakul, Emre Zafer, Özgür Deniz Turan

**Affiliations:** 1 Aydın Adnan Menderes University, Faculty of Medicine, Department of Obstetrics and Gynecology, Aydın, Turkey

**Keywords:** Abdominal incision, adnexal mass, pregnancy, case report

## Abstract

Adnexal torsion in pregnancy is a rare gynecologic emergency that causes severe abdominal pain. The current paper reports a case of a woman with 18 weeks pregnancy who was referred to our tertiary clinic with sudden lower abdominal pain. Ultrasound scan showed a very large multicystic adnexal torsion mass on the right side displacing the gravid uterus to the anterior left. An oblique paramedian incision was made for right salpingo-oophorectomy. To our knowledge the incision presented in this case has not been described in the literature previously. We suggest an alternative incision to be used during pregnancy especially for emergencies due to an adnexal mass.

## Introduction

Adnexal torsion is one of the most frequent non-obstetric emergencies requires surgical intervention during pregnancy. Fortunately, it develops only 1 in 5000 of pregnancies [1]. Adnexal torsion may occur in any trimester of pregnancy. The enlarged pregnant uterus may change the position of adnexal mass and the mass itself may be disposed as a result of torsion. Therefore, an adnexal mass with torsion may not be in its previously expected location. The effect of surgical technique on the growing pregnancy should be considered in choosing laparotomy technique and type and length of incision. The current paper aims to present a new laparotomy incision for urgent adnexal mass surgeries in pregnant patients.

## Patient and observation

**Figure 1:**
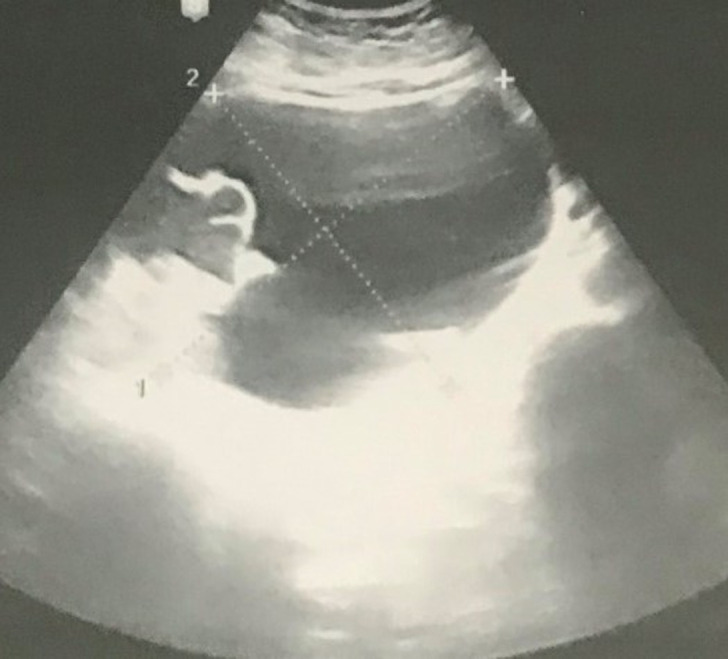
multicystic appearance of right adnexal mass, 15x12 cm

A 26-year-old pregnant patient with a history of two previous cesarean deliveries was referred to our tertiary clinic. The patient was having severe right sided abdominal pain with sudden onset. She was in the 18^th^ week of her pregnancy based on last menstrual period. Obstetric ultrasound measurements revealed a live pregnancy compatible with 18 weeks gestation without any abnormality. However, a 15x12cm in size edematous multicystic adnexal mass occupying the mid and upper right abdomen without vascularity was identified. The mass was suspected to originate from right ovary. It was pushing the pregnant uterus into the left and anterior direction (Figure 1). There was no ultrasonographic evidence of malignancy. Considering the severe sudden pain complaint and adnexal mass finding, explorative laparotomy was planned for the suspected diagnosis of adnexal torsion.

**Figure 2:**
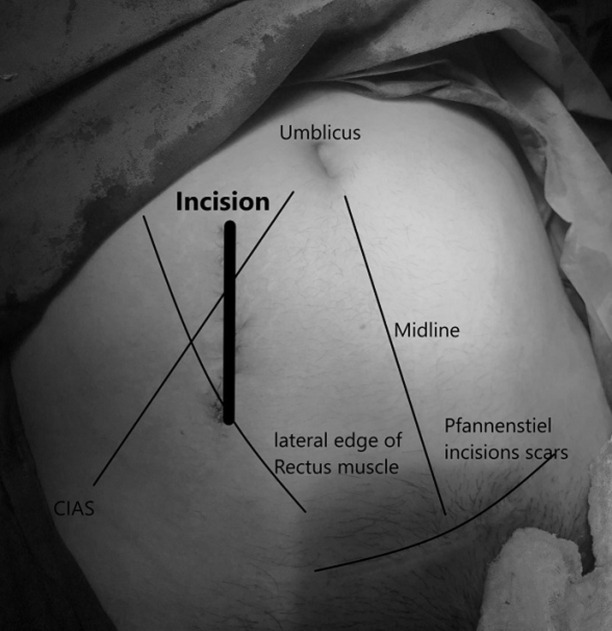
anatomic landmarks and the novel oblique abdominal incision (bold line) for adnexal pathologies in pregnancy; CIAS: Crista iliaca anterior superior

A mediolateral (near vertical) skin incision was made originated from infra-umbilical level on the lateral side of right rectus muscle. Specifically, the starting point for this slightly oblique incision was at 3cm lateral to the midline and near to lateral one third of the rectus muscle (Figure 2). The skin and fascia incisions were approximately 4cm and 2.5cm in length, respectively and this difference was due to the thick subcutaneous fat tissue. The oblique paramedian incision of the anterior rectus sheath was approximately 2cm medial to the lateral border of the rectus muscle and it was extended to caudal-lateral direction, down to 1/2cm to the lateral border of rectus muscle. The lateral edge of anterior rectus sheath incision was dissected off from the rectus muscle laterally up to semilunar line of rectus muscle in 1cm width. Then, the rectus muscle was retracted medially to expose the posterior sheath of rectus (below the arcuate line, only the transversalis fascia and parietal peritoneum are exposed).

An oblique incision was made on the posterior sheath of rectus and peritoneum behind the rectus muscle, in a projection of the anterior sheath incision. In this manner, the abdominal wall incisions were located at anterior and posterior sheaths of rectus muscle at least 1/2cm medial to the semilunar line and to the lateral border of rectus. In other words, incision was not made from fascial layers´ merging point or by muscle splitting dissection. Therefore, traction forces from enlarging uterus in advancing pregnant abdomen could be minimized. A large edematous, necrotic and dark purple torsion of a right adnexal mass was observed. It was pulled out and was partially dissected from its surface layer following a 2cm incision. The cystic structure was aspirated and right salpingo-ooferectomy was performed. The operation was completed by avoiding the manipulation of the pregnant uterus. Frozen section pathology of the removed specimen was reported as benign (necrotic tissues, teratoma) and final pathology was mucinous cystadenoma. Follow up on the pregnant patient was uneventful a month later.

## Discussion

The presented novel incision has a potential for rapid and shortcut entry into the abdomen to determine surgical diagnosis in pregnancy when needed, without a concern for touching the pregnant uterus. We preferred this new oblique incision to stay in front and behind of the rectus muscle. While applying this type of incision, the anterior sheath incised first, then rectus muscle is retracted sideway and then posterior sheath is incised too. Therefore the peritoneal cavity is easily accessed after fascial incision without any injury to rectus muscle. In pregnancy, having less concern about the traction forces on an incision by enlarging uterus would be an advantage. With this small incision, the pregnant abdominal wall may adapt to distension better as uterus enlarges. Another advantage of this incision would be ability to reach both mass and ovarian ligament for excision and detorsion of adnexa and/or the mass out of the abdomen.

In fact, this is not a lateral paramedian, pararectus or a muscle splitting incision. The choice of the incision type depends on the pathology requires direct access for surgical treatment. The type and the location of the incisions are selected based on its capacity for direct access to the presumed diagnosis requiring surgical treatment. Besides, the length of the incision must be adequate for surgical procedure and be easy to extend without disturbing abdominal wall function. This proposed new incision is placed at the periumbilical area and it may be placed at a point where operator could reach to the adnexal pathology instantly. Therefore, the incision depends on the uterine size, namely gestational week and location of the intraperitoneal pathology. It usually crosses the medial one third of the line between the umbilicus and anterior superior iliac spine on either side.

The starting point of the incision can easily be decided by ultrasound or physical examination considering adnexal mass size and gestational week to reach both mass and the utero-ovarian ligament. Smaller the mass size, smaller the incision a 2-3cm anterior rectus sheath incision should be enough. Care must be taken about inferior epigastric vessels as those may be exposed while retracting the rectus muscle medially and dissecting posterior sheath from the muscle. Instead of this incision we might cut through previous caesarean scar but it would be a longer incision and less adnexal mass exposure and the rule “do not touch the pregnant uterus” might be broken. A paramedian incision might not be appropriate as adnexa could be located far lateral to reach even though it may be favorable for lower risk of hernia when compared to midline incisions [2]. On the other hand, paramedian or pararectus incisions may have some other disadvantages such as nerve injury resulting in rectus muscle paralysis [3].

Seemingly, the only disadvantage of this proposed incision is not being parallel to Langer´s lines. Still, it does not extend to more than two dermatomes, T10 and T11 if not only in one. Postoperatively we did not observe any abdominal wall weakness or cosmetic complaint. If we had chosen a parallel incision, it might have prevented an extension option when needed. Laparoscopic approach may also possible in the first trimester or particularly for small size masses. The presented incision should be remembered as an alternative approach, especially in obese and in the second half of pregnancy with the advantage of almost a single 2-3cm incision without any hesitation about postoperative traction forces by created by enlarging uterus.

## Conclusion

To the best of our knowledge, the type of incision presented here has not been described previously [4]. We have two other adnexal torsion cases in the first and third trimester of their pregnancies that were operated by applying this same new type of incision. Later, both of them had term deliveries without any complications. With our limited practice, we aimed to share our experience of an alternative incision to be used during pregnancy especially for emergencies due to an adnexal mass.
